# Warts

**Published:** 1857-12

**Authors:** 


### WARTS.

If they give you no special inconvenience, let them alone. But if it is of essential importance to get rid of them, purchase half an ounce of *muriatic acid*, put it in a broad-bottomed vial, so that it will not easily turn over; take a stick as large as the end of a knitting-needle, dip it into the acid, and touch the top of the wart with whatever of the acid adheres to the stick; then, with the end of the stick, rub the acid into the top of the wart, without allowing the acid to touch the well skin. Do this night and morning, and a safe, painless, and effectual cure is the result.

